# Construction and validation of a deep learning-based diagnostic model for segmentation and classification of diabetic foot

**DOI:** 10.3389/fendo.2025.1543192

**Published:** 2025-04-09

**Authors:** Guang-Xin Zhou, Yu-Kun Tao, Jin-Zheng Hou, Hui-Juan Zhu, Li Xiao, Na Zhao, Xiao-Wen Wang, Bao-Lin Du, Da Zhang

**Affiliations:** ^1^ Department of Endocrinology, Air Force Medical Center, Air Force Medical University, Beijing, China; ^2^ Chongqing Zhijian Life Technology Co., Ltd, Chongqing, China

**Keywords:** diabetic foot ulcers, artificial intelligence, deep learning, segmentation, classification

## Abstract

**Objective:**

This study aims to conduct an in-depth analysis of diabetic foot ulcer (DFU) images using deep learning models, achieving automated segmentation and classification of the wounds, with the goal of exploring the application of artificial intelligence in the field of diabetic foot care.

**Methods:**

A total of 671 images of DFU were selected for manual annotation of the periwound erythema, ulcer boundaries, and various components within the wounds (granulation tissue, necrotic tissue, tendons, bone tissue, and gangrene). Three instance segmentation models (Mask2former, Deeplabv3plus, and Swin-Transformer) were constructed to identify DFU, and the segmentation and classification results of the three models were compared.

**Results:**

Among the three models, Mask2former exhibited the best recognition performance, with a mean Intersection over Union of 65%, surpassing Deeplabv3’s 62% and Swin-Transformer’s 52%. The Intersection over Union value of Mask2former for wound recognition reached 85.9%, with IoU values of 80%, 78%, 62%, 61%, 47%, and 39% for granulation tissue, gangrene, bone tissue, necrotic tissue, tendons, and periwound erythema, respectively. In the wound classification task, the Mask2former model achieved an accuracy of 0.9185 and an Area Under the Curve of 0.9429 for the classification of Wagner grade 1-2, grade 3, and grade 4 wounds.

**Conclusion:**

Among the three deep learning models, the Mask2former model demonstrated the best overall performance. This method can effectively assist clinicians in recognizing DFU and segmenting the tissues within the wounds.

## 1 Introduction

Diabetic foot ulcer (DFU) is one of the most severe and costliest chronic complications of diabetes, with approximately 25% to 34% of diabetic patients experiencing at least one episode of DFU in their lifetime. If not treated promptly and appropriately, diabetic foot can lead to severe consequences, including amputation and even death (1). Systematic reviews suggest that the median delay for referral of DFU patients from primary care specialists is between 7 to 31 days, while the median time to initiate final treatment ranges from 6.2 to 56 days (2). In China, 80.8% to 95.6% of DFU patients face delays in seeking medical care, with the average delay extending to 54.81 days (3). The complexity of diabetic foot cases, the shortage of specialized healthcare professionals, and the lack of experience in wound assessment at primary healthcare institutions present significant challenges for the early diagnosis and treatment of diabetic foot. As the population of diabetic patients continues to grow, enhancing the management of DFU has become increasingly urgent.

DFU of varying severity exhibit numerous visual characteristics, such as granulation tissue, necrotic tissue, erythema, tendons, bones, and gangrene. Traditional computer vision algorithms based on machine learning primarily rely on multiple stages, including feature sensing, image preprocessing, feature extraction, feature selection, and inference prediction, to identify abnormal regions (4, 5). These methods utilize differences in color and texture descriptors on the surface of abnormal areas (e.g., wounds) and employ classifiers such as support vector machines, neural networks, random forests, or Bayesian classifiers to perform binary classification (i.e., distinguishing between healthy skin and ulcerated skin) (6–10). In recent years, with the rapid advancement of computer vision technology, deep learning has demonstrated exceptional effectiveness in processing DFU images. Han et al. (11)enhanced the Faster Region-based Convolutional Neural Network algorithm using K-means clustering to achieve automatic recognition and localization of diabetic foot wounds according to Wagner grades. Goyal et al. (12) proposed a deep learning-based method for real-time detection and localization of DFUs. Huang HN et al. (13), through transfer learning and the Faster R-CNN algorithm, were able to perform image segmentation, distinguishing between ulcers, sutures, and gangrene caused by vascular blockage in DFU, achieving up to 90% accuracy in wound image detection. Zhao Nan et al. (14) conducted a study involving 1,042 images of DFU, with manual annotation of ulcer boundaries and different color regions, achieving wound localization and area measurement.

Deep learning-based artificial intelligence (AI) technology has shown great potential in the image recognition and lesion area segmentation of DFU, providing a powerful tool to enhance patient management and potentially improve the current challenges in diagnosis and treatment. However, existing visual computing research has largely focused on DFU recognition, wound segmentation, and distinguishing between infected and non-infected areas, while neglecting critical features such as tendons and bones within the wound, which directly influence the grading of diabetic foot. Additionally, current approaches have not achieved simultaneous recognition of tissue classification and infection characteristics. This study aims to explore the optimization of deep learning techniques for semantic segmentation of wound and surrounding tissue areas, and to construct models for wound feature recognition and grading diagnosis, with the goal of developing an AI-based tool to assist in the recognition of diabetic foot.

## 2 Material and methods

### 2.1 Patients and images

A total of 671 images of DFU were collected from patients treated at the Air Force Medical Center between January 2015 and December 2023. The diagnostic criteria for diabetic foot include patients with newly diagnosed diabetes or a history of diabetes, presenting with infections, ulcers, or tissue destruction in the foot, usually accompanied by lower limb neuropathy and/or peripheral arterial disease (15). The inclusion criteria for this study were as follows: images of Wagner grade 1-4 DFU confirmed by professional healthcare personnel; images clearly displaying the ulcer area of the foot with sufficient resolution to accurately distinguish tissue characteristics such as granulation tissue, necrosis, tendons, bone, and gangrene; and images sourced from various devices and conditions to enhance the robustness of the model. Exclusion criteria included: poor-quality images, such as those that are blurred, underexposed, or overexposed, or where key information is obscured; ulcers not caused by diabetes or of unclear etiology; incomplete image information with annotation errors or missing critical details; images of unknown origin or those involving copyright disputes; and any images that do not meet ethical and legal requirements.

### 2.2 Data preprocessing

The collected images underwent preprocessing to improve processing efficiency and accuracy. Given the substantial variability in original dimensions (ranging from 3864×5152 to 1080×1920 pixels), we prioritized scaling over cropping to preserve critical anatomical details and maintain automation. Bilinear interpolation was employed for resizing: inserting new pixels via interpolation during upscaling and averaging neighboring pixels during downscaling. All images were standardized to 1024×1024 pixels through this scaling approach.

**Figure 1:**
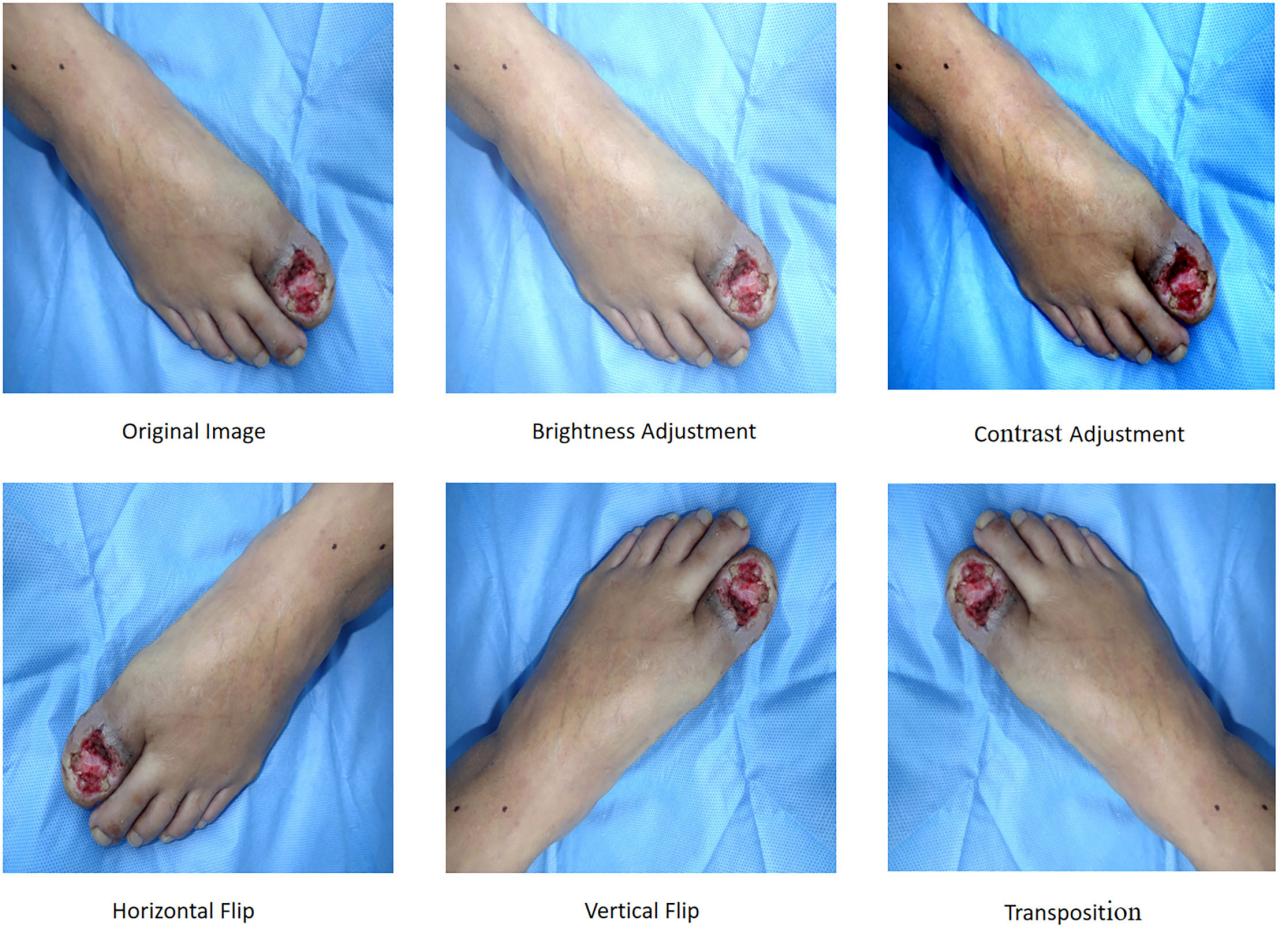
Data augmentation techniques.

To enhance model robustness, we implemented five data augmentation techniques in **Figure 1**:

1. Brightness Adjustment: Modifying the R, G, B channel values of pixels to alter overall image brightness.
2. Contrast Adjustment: Enhancing the difference between the brightest and darkest regions to improve image hierarchy and visual impact.
3. Horizontal Flip: Mirroring the image along the vertical axis.
4. Vertical Flip: Mirroring the image along the horizontal axis.
5. Transposition: Swapping rows and columns to invert the image spatially.

### 2.3 Dataset for the study

A total of 671 DFU images were collected, with annotation work conducted by an expert team from the Endocrinology Department of the Air Force Medical Center. These physicians, who have extensive experience in diabetic foot care, used Labelme software for precise annotation. The annotations included the ulcer area, granulation tissue, necrotic tissue, tendons, bone, gangrene, and infection across seven categories. Specifically, 631 images included granulation tissue, 458 included necrotic tissue, 130 included tendons, 140 included bone, 122 included gangrene, and 264 included infection. This study utilized the Wagner classification system (16), and considering the similarity in treatment strategies between ulcers affecting the skin and soft tissue layers in Wagner grades 1 and 2, we combined Wagner grades 1 and 2 into a single category. This adjustment aimed to more precisely reflect the characteristics of the wounds and the extent of tissue damage while ensuring clear performance in the model’s classification tasks:

W1-2 Grade: Wounds limited to the skin and soft tissue, without bone involvement or abscess formation.
W3 Grade: Wounds have invaded deeper tissues, manifested by bone exposure and/or tendon damage, but gangrene has not yet occurred.
W4 Grade: Wounds accompanied by gangrene, indicating tissue necrosis beyond the capacity for repair.

### 2.4 Deep learning procedure

**Figure 2:**
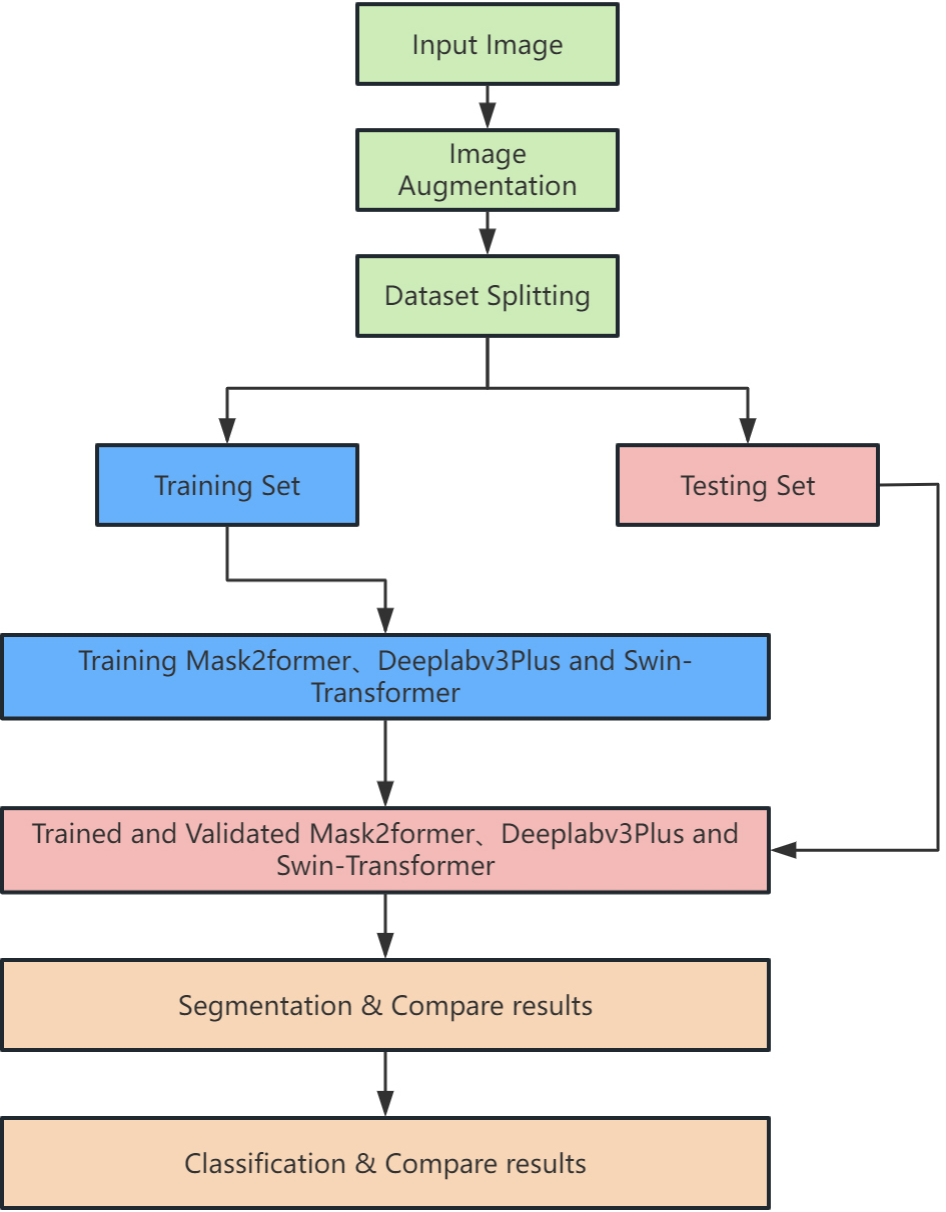
Schematic diagram of the study flow.

In this study, we employed three different deep learning models: Deeplabv3Plus, Swin-Transformer, and Mask2Former, chosen for their distinct advantages in image segmentation tasks. Deeplabv3Plus is a well-established model based on convolutional neural networks (CNNs) with widespread application in semantic segmentation (17); Swin-Transformer leverages the Transformer architecture to handle long-range dependencies, making it suitable for recognizing complex features (18); and Mask2Former combines the global modeling capabilities of Transformers with the local perception abilities of CNNs, making it particularly effective for multi-label recognition tasks (19). Deeplabv3Plus uses the CNNs model ResNet101 as its backbone, while Swin-Transformer and Mask2Former use Transformer models based on self-attention mechanisms as their backbone networks. CNNs have translation invariance and local connectivity, primarily extracting local features in images through convolution and pooling operations, whereas Transformer network models utilize self-attention mechanisms and multi-layer perception structures to capture global feature representations through complex spatial transformations and long-range feature dependencies. These models were pre-trained on the publicly available ImageNet dataset (20), with the pre-trained model parameters used as initialization parameters. The collected DFU images were then used to fine-tune the models, with training progress monitored through loss function values, accuracy curves on the training and test sets, and other metrics until the loss values were sufficiently low and the accuracy curves stabilized. The overall workflow of the study is illustrated in **Figure 2**.

### 2.5 Evaluation of the performance of deep learning models

To quantitatively assess the performance of the proposed models, the DFU image set was divided into two subsets in an 8:2 ratio, used for model training and testing, respectively. We employed two key metrics: Intersection over Union (IoU) and Dice coefficient to comprehensively evaluate the recognition and segmentation performance of the three deep learning models on DFU images. Both metrics quantify the overlap between the predicted ulcer area by the model and the ground truth area annotated by experts. A perfect match would yield IoU and Dice coefficients of 1, indicating complete concordance between the predicted and actual areas; conversely, values of 0 would indicate no overlap. In addition to evaluating the models’ performance on segmentation tasks through IoU and Dice coefficients, we also assessed the diabetic foot Wagner grading task using metrics such as precision, sensitivity, specificity, accuracy, F1 score, area under the curve (AUC), and receiver operating characteristic (ROC) curve. The specific calculations for each metric are detailed in **Table 1**.

**Table 1:** Calculation formulae.

Metric	Formula	Description
Precision	$\frac{\text{TP}}{\text{TP}+\text{FP}}$	Correct positive predictions over all positive predictions
Sensitivity	$\frac{\text{TP}}{\text{TP}+\text{FN}}$	Fraction of correct positive predictions
Specificity	$\frac{\text{TN}}{\text{TN}+\text{FP}}$	Fraction of correct negative predictions
Accuracy (ACC)	$\frac{\text{TP}+\text{TN}}{\text{TP}+\text{FP+TN}+\text{FN}}$	Correct prediction ratio
F1 Score	$\frac{2TP}{2TP+\text{FP+FN}}$	The harmonic mean of precision and recall
AUC (area under the curve)	$\frac{\text{sn+sp}}{2}$	Threshold-invariant prediction quality
DICE	$\frac{2TP}{2TP+\text{FP+FN}}$	Coefficient for measuring overlap between predicted and actual regions. Similar to F1 score
IoU	$\frac{\text{TP}}{\text{TP}+\text{FP+FN}}$	Another coefficient for measuring the overlap between predicted and actual regions

sn, Sensitivity; sp, Specificity; TP, True Positives; FN, False Negatives; FP, False Positives; TN, True Negatives.

### 2.6 Hardware and software specifications

The training server used in this experiment was configured with the CentOS Linux Release 7.4.1708 operating system, Intel(R) Xeon(R) CPU, Nvidia GeForce RTX 3090 GPU, and CUDA Version 11.4. The AI framework was implemented using the Pytorch framework, with OpenCV version 4.3.0.36.

## 3 Result

### 3.1 Model construction and comparison

#### 3.1.1 Training and inference efficiency

During 100 epochs of training, the DeeplabV3Plus model exhibited faster training speed, completing the entire process in approximately 11 hours, with an inference time of around 0.19 seconds per image. The Swin-Transformer model required a slightly longer training period of about 14 hours and 30 minutes for 100 epochs, with an inference time of 0.42 seconds. The Mask2Former model also had an extended training period, taking 13 hours and 10 minutes, which is attributable to its complex architecture and focus on detailed features; however, its inference time was only 0.39 seconds, indicating that despite the longer training time, the model is capable of delivering efficient real-time performance.

#### 3.1.2 Wound segmentation performance

**Figure 3:**
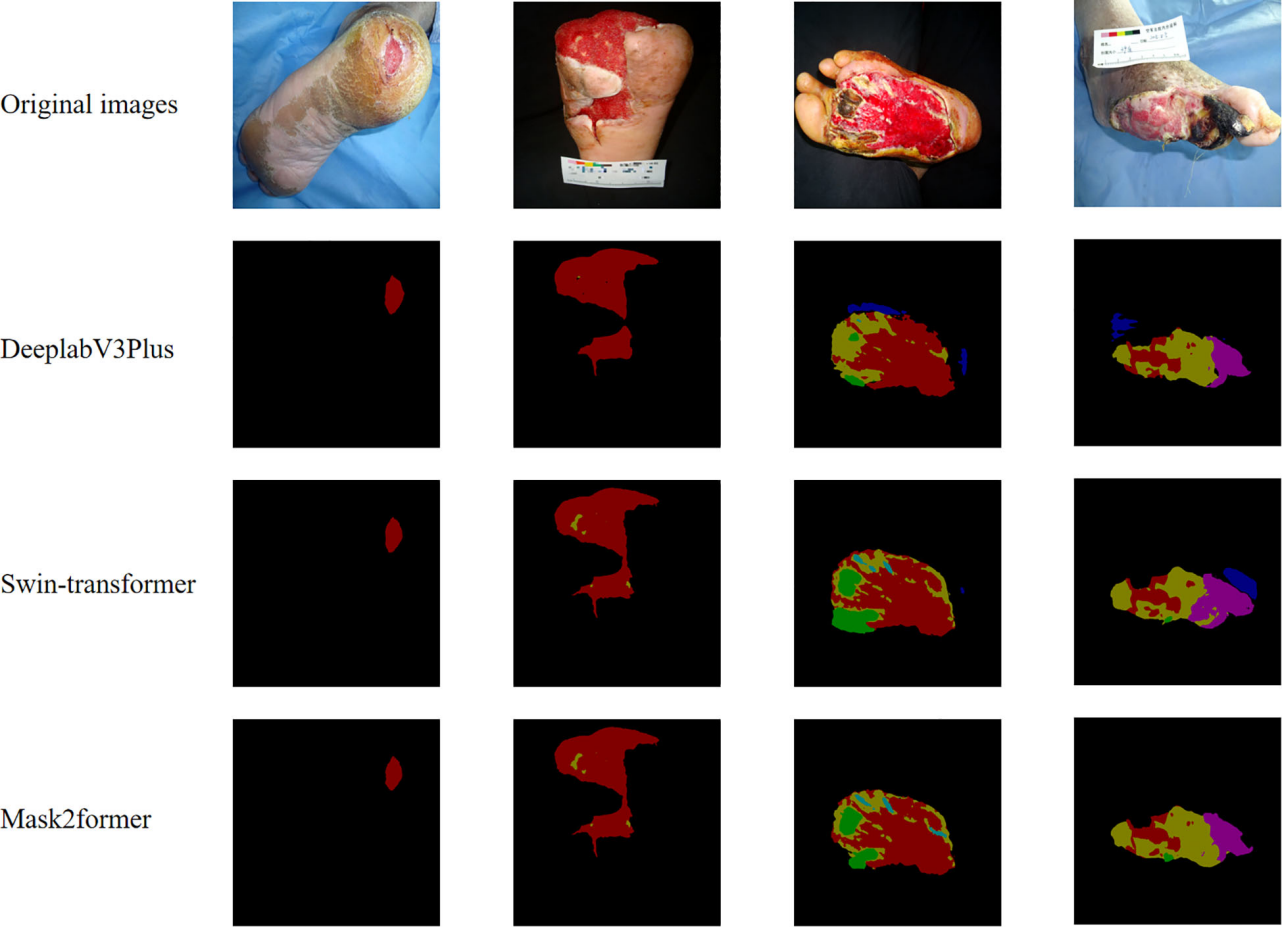
Demonstration of three model segmentation tasks. Granulation (red), Necrotic tissue (yellow), Bone (green), Tendon (cyan), Infection (blue), Gangrene (purple).

The segmentation results of the three models on DFU are presented in **Table 2** and **Figure 3**. In the wound segmentation task, the IoU value for the Mask2Former model was 85.88%, closely matching Swin-Transformer’s 85.9%. However, Mask2Former outperformed in multi-label recognition tasks, achieving IoU values of 78.78%, 59.53%, 48.72%, 53.45%, and 77.14% for granulation tissue, necrotic tissue, tendons, bone, and gangrene, respectively. Regarding the mean Intersection over Union (mIoU) on the test set, Mask2Former achieved 65.79%, Swin-Transformer 65.31%, and DeeplabV3Plus 52.86%. These results indicate that while Mask2Former and Swin-Transformer performed comparably in single wound segmentation, Mask2Former demonstrated a clear advantage in multi-label recognition tasks.

**Table 2:** Segmentation identification results of the three models in diabetic foot wounds:.

Model	Indicator	Wound	Infection	Granulation	Necrosis	Tendon	Bone	Gangrene
DeeplabV3Plus	IoU	78.48	45.45	65.77	39.12	26.20	32.25	64.22
	ACC	84.72	61.06	79.93	49.14	27.45	37.51	69.41
	Dice	87.94	62.50	79.35	56.23	41.52	48.77	78.21
	mIoU	52.86						
	mAcc	60.46						
	mDice	66.44						
Swin-transformer	IoU	87.36	47.22	78.52	59.18	47.95	48.76	77.84
	ACC	92.71	59.5	88.19	73.26	58.63	73.12	85.85
	Dice	93.25	64.15	87.97	74.35	64.82	65.56	87.54
	mIoU	65.31						
	mAcc	76.80						
	mDice	77.60						
Mask2former	IoU	85.88	45.14	78.78	59.53	48.72	53.45	77.14
	ACC	89.60	50.62	86.67	72.0	63.12	76.61	89.31
	Dice	92.40	62.20	88.13	74.63	65.52	69.66	87.09
	mIoU	65.79						
	mAcc	76.84						
	mDice	78.00						

#### 3.1.3 Infection recognition ability

**Figure 4:**
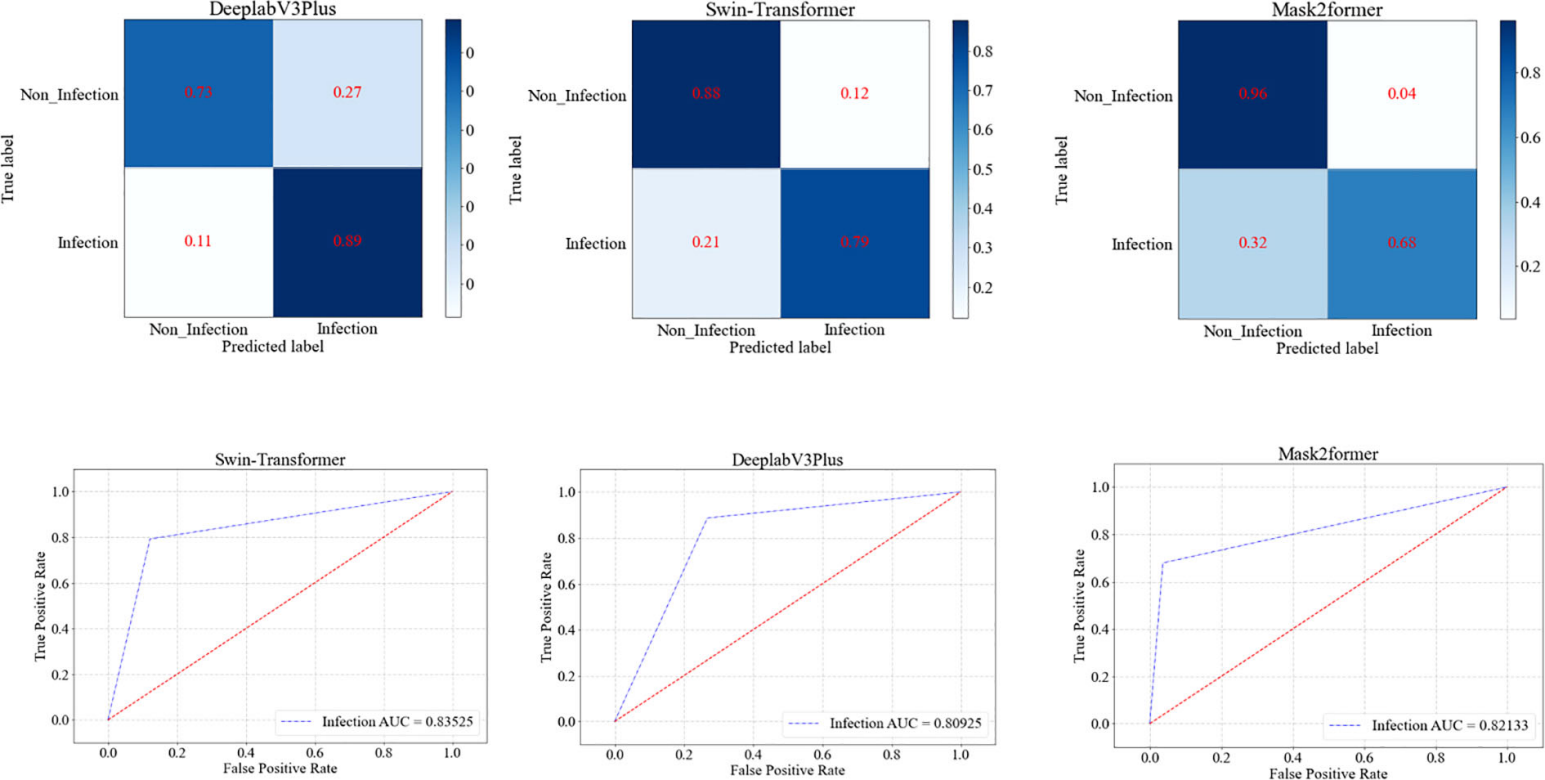
ROC curves of the three models for identifying the presence of infection in diabetic wounds.

In our study, we compared the performance of the three deep learning models in identifying the presence or absence of infection in diabetic foot wounds (**Table 3**, **Figure 4**). The DeeplabV3Plus model exhibited a sensitivity of 0.8868 in infection state recognition but was slightly weaker in specificity, with a value of 0.7317, resulting in an overall accuracy of 0.7926 and an Area Under the Curve (AUC) of 0.8093. The Swin-Transformer model showed balanced performance in recognizing both infected and non-infected states, with a sensitivity of 0.7925 and a specificity of 0.8780, achieving an accuracy of 0.8444 and an AUC of 0.8353. The Mask2Former model performed best across all evaluation metrics, particularly in accuracy and AUC, where it achieved 0.8519 and 0.8353, respectively.

**Table 3:** Results of the three models in recognizing the presence or absence of infection in diabetic foot wounds:.

Model	Class	Precision	Sensitivity	Specificity	F1	ACC	AUC
DeeplabV3Plus	infections	0.6812	0.8868	0.7317	0.7705	0.7926	0.8093
	non-infectious	0.9091	0.7317	0.8868	0.8108		
Swin-transformer	infections	0.8077	0.7925	0.8780	0.8000	0.8444	0.8353
	non-infectious	0.8675	0.8780	0.7925	0.8727		
Mask2former	infections	0.9231	0.6792	0.9634	0.7826	0.8519	0.8353
	non-infectious	0.8229	0.9634	0.6792	0.8876		

#### 3.1.4 Grading diagnosis of DFU

**Figure 5:**
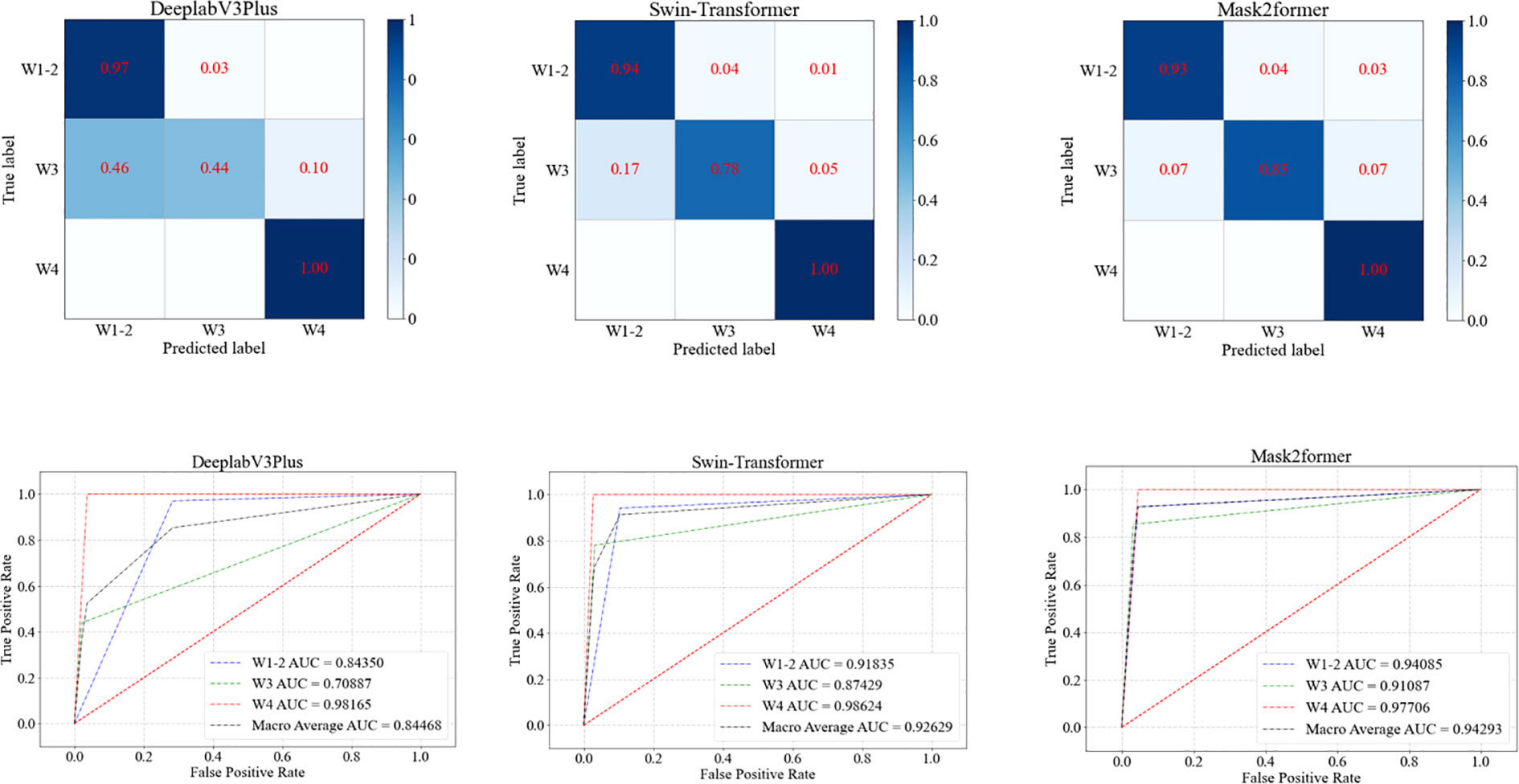
ROC curves of three models for grading diabetic foot wounds.

In the task of grading DFU, the Mask2Former model demonstrated the best overall performance (**Table 4**, **Figure 5**). The Mask2Former model exhibited high precision and specificity across all grade classification tasks and achieved the best results in overall accuracy (ACC) and AUC, with ACC at 0.9185 and AUC at 0.9429. The DeeplabV3Plus model achieved corresponding values of 0.8148 and 0.8447, while the Swin-Transformer model had an ACC of 0.9037 and an AUC of 0.9263. These results highlight the superior performance of the Mask2Former model in the grading diagnosis of DFU.

**Table 4:** Results of the three models in grading diabetic foot wounds:.

Model	Disaggregated indicators	Precision	Sensitivity	Specificity	F1	ACC	AUC
DeeplabV3Plus	W1-2	0.7765	0.9706	0.7164	0.8628	0.8148	0.8447
	W3	0.9	0.439	0.9787	0.5901		
	W4	0.8667	1.0	0.9633	0.9286		
Swin-transformer	W1-2	0.9014	0.9412	0.8955	0.9209	0.9037	0.9263
	W3	0.9143	0.7805	0.9681	0.8421		
	W4	0.8966	1.0	0.9725	0.9455		
Mask2former	W1-2	0.9545	0.9265	0.9552	0.9403	0.9185	0.9429
	W3	0.9211	0.8537	0.9681	0.8861		
	W4	0.8387	1.0	0.9541	0.9123		

## 4 Discussion

This study successfully constructed and compared the performance of three deep learning models (Mask2Former, DeeplabV3Plus, and Swin-Transformer) in the recognition and segmentation of DFU images. The results revealed the significant advantages of the Mask2Former model in terms of mean accuracy, segmentation precision, and grading diagnosis, achieving effective AI-based wound feature recognition and Wagner grading.

Previous studies have focused on segmenting and identifying the extent of the wound area and structural regions within DFU using deep learning. Can Cui et al. (21)proposed a CNN-based method for precise wound area segmentation, achieving an accuracy of 72%. The 2022 DFU Segmentation Challenge (22)showcased the latest advancements in DFU segmentation, where the winning team achieved a Dice score of 0.7287 in the wound recognition task. Our results indicate that the Mask2Former model achieved an IoU of 85.88% in wound segmentation tasks, and reached a Dice score of 0.9240, with the Swin-Transformer model achieving an even higher Dice score of 0.9325. Our models offer superior accuracy in wound segmentation. The core innovation of the Mask2Former model lies in its mask attention mechanism, which enhances the precision and efficiency of local feature extraction by focusing on and optimizing cross-attention processing within the predicted mask region. Notably, the unified architecture and ease of training of Mask2Former enable it to effectively adapt to DFU image recognition without requiring specific adjustments (19).

Compared to earlier studies (13, 14, 23, 24), we have achieved significant progress in the accuracy of tissue recognition within diabetic foot wounds. Our study not only accomplished precise segmentation and delineation of the wound area but also extended to the identification of necrotic tissue, tendons, bone, and gangrene within the wound. Detailed wound segmentation and tissue recognition are critical steps in managing DFU. By accurately distinguishing between granulation tissue, necrotic tissue, tendons, bone, gangrene, and the periwound erythema, we can provide a clear basis for ulcer severity grading, as well as offer precise information for prognosis assessment and treatment planning. For instance, the presence of necrotic tissue indicates the need for debridement. Exposed bone is at risk of osteomyelitis, which can increase the likelihood of amputation, prolong hospitalization, extend antibiotic treatment duration, and delay healing (25).

Diabetic foot infection (DFI) is the most common reason for hospitalization among patients with diabetic-related foot ulcers and is a major cause of lower limb amputation. Only about 46% of infected foot ulcers heal within a year (with 10% recurring), and 15% of patients die, while 17% require lower limb amputation (26). Goyal M et al. (27)developed a novel dataset and superpixel color descriptor technique combined with an ensemble CNN model, effectively improving the recognition efficiency of ischemia and infection, achieving an infection recognition accuracy of 73%. Yogapriya J et al. (28)constructed the Diabetic Foot Infection Network (DFINET), specifically designed to assess infection and non-infection states in DFU images, with a recognition accuracy of 91.98%. In this study, the Mask2Former model achieved an infection recognition accuracy (ACC) of 0.8519 and an AUC of 0.8353. Previous studies have primarily focused on identifying the presence of infection, but delineating the extent of infection is not only essential for distinguishing between mild, moderate, and severe infections but also directly influences the choice of antibiotics and treatment duration (29). This study also developed a model for determining the extent of diabetic foot infection, although model performance still needs improvement. This may be due to image quality and the variability in how annotators define the extent of infection.

Diabetic foot prognosis is closely linked to Wagner grading. In this study, AI-based wound feature recognition was integrated with the Wagner system to achieve automated grading. Notably, Wagner grade 5 cases (characterized by extensive necrosis and requiring urgent surgical intervention) were excluded, as their management relies on comprehensive clinical evaluation rather than image analysis alone. The Mask2Former model demonstrated robust performance in distinguishing Wagner grades 1–4, with AUC values of 0.97 (grades 1–2), 0.82 (grade 3), and 0.78 (grade 4). This AI-assisted approach addresses critical challenges in diabetic foot care—including limited expertise and time constraints—by providing efficient, objective decision support for severity assessment and treatment prioritization.

While our study advances wound feature recognition and grading in DFUs, several limitations warrant attention:

1. Narrow applicability: The model focuses solely on DFU wound characterization and lacks utility for differential diagnosis of non-diabetic foot lesions.
2. Data constraints: The pilot-scale dataset (n=671 images) and absence of external validation limit generalizability. Future work will expand sample diversity and incorporate multi-center data.
3. Image standardization: Variability in image quality (e.g., lighting, angles) may impair model robustness. Standardized imaging protocols and scale integration will enhance segmentation accuracy and enable wound area quantification.
4. Infection localization: While infection presence detection is reliable, precise spatial delineation requires improved annotation strategies and advanced algorithms.
5. Clinical integration: Augmenting image analysis with clinical data (e.g., vascular status, wound location) could refine prognostic predictions and personalize treatment plans.

The Mask2Former model achieves high-precision segmentation and classification of DFUs, excelling in identifying critical tissue components (necrotic tissue, tendons, bone) and Wagner grading. By automating severity assessment, it addresses clinical challenges such as diagnostic delays and expertise shortages, particularly in resource-limited settings. These capabilities lay the groundwork for intelligent DFU management systems, supporting telemedicine and real-time monitoring to optimize patient outcomes.

## Data Availability

The raw data supporting the conclusions of this article will be made available by the authors, without undue reservation.
